# Molecular Processes in Chondrocyte Biology

**DOI:** 10.3390/ijms21114161

**Published:** 2020-06-11

**Authors:** Toshihisa Komori

**Affiliations:** Basic and Translational Research Center for Hard Tissue Disease, Nagasaki University Graduate School of Biomedical Sciences, Nagasaki 852-8588, Japan; komorit@nagasaki-u.ac.jp; Tel.: +81-95-819-7637; Fax: +81-95-819-7638

Chondrocyte biology is a hot topic, because osteoarthritis (OA) is a serious problem in an aging society, but there are no fundamental therapeutic drugs. This Special Issue aimed to collect the recent progress in the molecular aspects of chondrocyte proliferation, differentiation, and death and the pathogenesis of OA and rheumatoid arthritis (RA). 

In the comprehensive review of skeletal development, Shawn A. Hallett and colleagues emphasized skeletal stem cells, which are a population of chondrocytes in the resting zone expressing parathyroid hormone-related protein (PTHrP). These cells have an ability of self-renewal and give rise to columnar chondrocytes. Further, these cells transdifferentiate into osteoblasts and bone marrow stromal cells during skeletal development. Lineage-tracing experiments revealed the transdifferentiation of terminally differentiated chondrocytes into osteoblasts and bone marrow stromal cells and identified PTHrP-expressing skeletal stem cells and transient mesenchymal precursors (borderline chondrocytes) [1]. Hironori Hojo and Shinsuke Ohba reviewed the gene regulatory mechanisms addressed by next-generation sequencer analyses, which included histone modification, binding of transcription factors and cofactors, and the topological organization of chromatin, focusing on Sox family genes (*Sox9*, *Sox5*, and *Sox6*) in the transcriptional regulation of chondrocyte-distinct genes. For example, the binding of Sox9 and AP-1 or Gli to the enhancers and their interaction with the target genes regulate chondrogenesis [2]. Satoshi Kubota and colleagues described the basal mechanisms of genome editing by retrotransposons and their involvement in skeletal development. For example, the mutation of *Poc1a*, which encodes a component of centrosome, by long interspersed nuclear element-1 (LINE-1)-mediated retrogene transposition leads to the craniofacial and skeletal abnormalities through the impaired polarity of growth plate chondrocytes. Short-legged dogs were created by the addition of extra fibroblast growth factor 4 (*FGF4*) cDNA, which is caused by LINE-1. The integration of *Alu* retrotransposon to *ZBTB38* is involved in the determination of height in humans [3]. 

Mitsuhiro Hoshijima and colleagues showed the interaction between CCN2 and Rab14, their colocalization in the cytosol, and the reduction in the extracellular proteoglycan by dominant-negative Rab14 and suggested that this association may be involved in the trafficking of proteoglycan-containing vesicles in chondrocytes [4]. Our group published two reports related to chondrocyte proliferation. Antxr1/Tem8 is a receptor for anthrax, and its mutation causes GAPO syndrome, which is characterized by growth retardation (G), alopecia (A), pseudoanodontia (P), and optic atrophy (O). Qing Jiang and colleagues showed that *Antxr1* expression is directly regulated by Runx2; it plays an important role in chondrocyte proliferation, and the overexpression of *Antxr1* increases chondrocyte proliferation but causes chondrocyte apoptosis accompanied by matrix mineralization [5]. Hck is a member of the Src tyrosine kinase family. Viviane K. S. Kawata Matsuura and colleagues showed that *Hck* is highly expressed in chondrocytes, and its expression is directly regulated by Runx2 and that the expression of a constitutively active form of *Hck* (*Hck*^CA^) disorganized the growth plate but markedly increased chondrocyte proliferation without enhancing apoptosis. Further, Hck^CA^ activated the Wnt and hedgehog signaling pathways, indicating that chondrocyte proliferation is increased by the activation of the Wnt and hedgehog signaling pathways, in addition to the well-known Src family protein signaling pathways, through Akt, ERK, and Stat [6].

Tsuyoshi Shimo and colleagues showed that IL-1β and RARγ are expressed at the fracture site, and IL-1β induces *Mmp13* and *Ccn2* expression through p38 MAPK, which is inhibited by an RAR inverse agonist, indicating an important role of IL-1β via p38 MAPK in the endochondral bone formation during fracture healing and the actions of an RAR inverse agonist as a modulator of this process [7]. Sonia A. Garcia and colleagues examined the effect of RARγ agonists on osteochondromas, which are cartilage-capped growths located proximate to the physis, and found that the RARγ agonist inhibits matrix synthesis, promotes cartilage matrix degradation, and stimulates cell death, implicating its antitumor function of osteochondromas [8]. Eijiro Jimi and colleagues reviewed the NF-κB signaling pathway. The classical NF-κB signaling pathway regulates chondrocyte apoptosis through the induction of BMP2, while the alternative NF-κB pathway regulates chondrocyte proliferation and differentiation. The deletion of p65 increases apoptosis and exacerbates OA, the heterozygous deletion of p65 suppresses OA development, and the activation of p65 induces the expression of matrix metalloproteinases (MMPs) and vascular endothelial growth factor (VEGF) through HIF2α and accelerates OA [9]. 

Riko Nishimura and colleagues overviewed the signal transduction pathways in OA, RA, fibrodysplasia ossificance progressive (FOP), and achondroplasia (ACH). Transcription factors, including Runx2, C-EBPβ, HIF2α, Sox4, and Sox11 and IL-1β through NF-κB, IκBζ, and the Zn2+-ZIP8-MTF1 axis, are involved in the onset and progression of OA. IL-1, IL-6, and tumor necrosis factor α (TNFα) play major roles in RA through the NF-κB and JAK/STAT pathways. FOP is caused by the mutation of *ACVR1*, and mTOR inhibitors inhibit the ectopic ossification. C-type natriuretic peptide can rescue ACH-like phenotypes through MAPK [10]. Jane Riegger and Rolf E. Brenner provided a comprehensive overview on posttraumatic OA. Cartilage trauma causes cell death and synovial inflammation and releases reactive oxygen species (ROS)/nitric oxide (NO) and damage-associated molecular patterns (DAMPs), which further trigger chondrocyte death, synovial response, and the production of catabolic enzymes and proinflammatory factors. OA chondrocytes show hypertrophy and senescence-like phenotypes, which also lead to chondrocyte death and the increased expression of cytokines and catabolic enzymes [11]. Chia-Chun Tseng and colleagues overviewed RA, especially focusing on chondrocytes as causable cells of RA pathogenesis. Chondrocytes directly participate in RA pathogenesis through the release of multiple cytokines and chemokines and the production of MMPs, prostaglandin E, vascular endothelial growth factor (VEGF), and NO. Noncoding RNA and many pathways, including necroptosis, pyroptosis, hedgehog, MAPK, JAK/STAT, AP-1, and JNK2, are involved in the pathogenesis of chondrocyte dysfunction [12]. Narjès Hafsia and colleagues evaluated the role of gelactin 3 (GAL3), which is important for the formation of primary cilia that are able to sense mechanical stress, in chondrocyte primary cilium formation and OA. *Gal3*^−/−^ mice had abnormal cilia and exhibited premature OA during aging and exacerbated joint instability-induced OA, demonstrating the importance of GAL3 in chondrocyte primary cilium formation and OA development [13]. 

Elisabetta Chiaradia and colleagues performed a proteome analysis of equine osteochondrosis, which is a joint disorder characterized by focal chondronecrosis and growing cartilage retention. The extracellular matrix, cytoskeletal, chaperone, cell adhesion, and signaling proteins were differentially expressed in chondrocytes from osteochondrotic cartilage compared with those from healthy cartilage [14]. Eriko Toyada and colleagues examined the efficiency of polydactyly-derived chondrocyte sheets using an orthotopic xenogeneic transplantation model, and the efficacy-correlated genes and proteins were identified by transcriptomic and proteomic analyses. MIA, DKK1, GREM1, and ESM1 exhibited correlations between the amount of secretion and efficacy [15]. Kentaro Homan and colleagues performed comprehensive glycomic analyses to examine the alterations of the levels of various glycans derived from glycoconjugates during chondrocyte maturation using primary chondrocytes. The cellular glycan alterations were closely associated with chondrocyte maturation [16]. 

I wish to express my appreciation to each of the authors for their contributions. The molecular aspects of chondrocyte proliferation, differentiation, and death and the pathogenesis of OA and RA are going to be revealed, but extensive investigations are still needed to establish a molecular basis and develop new therapies and drugs. 
